# Serological detection of *Rickettsia* spp. and evaluation of blood parameters in pet dogs and cats from Bangkok and neighboring provinces

**DOI:** 10.1371/journal.pone.0297373

**Published:** 2024-03-07

**Authors:** Chanon Fa-ngoen, Gunn Kaewmongkol, Natnaree Inthong, Ampai Tanganuchitcharnchai, Mohammad Yazid Abdad, Jarunee Siengsanan-Lamont, Stuart D. Blacksell, Sarawan Kaewmongkol

**Affiliations:** 1 Faculty of Veterinary Technology, Kasetsart University, Bangkok, Thailand; 2 Queen Saovabha Memorial Institute, Thai Red Cross Society, Bangkok, Thailand; 3 Faculty of Veterinary Medicine, Kasetsart University, Bangkok, Thailand; 4 Mahidol-Oxford Tropical Medicine Research Unit, Faculty of Tropical Medicine, Mahidol University, Bangkok, Thailand; 5 Centre for Tropical Medicine, Nuffield Department of Clinical Medicine, John Radcliffe Hospital, Oxford, United Kingdom; National Veterinary Research Institute (NVRI), NIGERIA

## Abstract

Rickettsiosis is caused by *Orientia* spp. and *Rickettsia* spp., arthropod-borne zoonotic intracellular bacteria. The close relationships between pet dogs, cats and owners increase the risk of rickettsial transmission, with limited studies on the seroprevalence in pets. This study investigated the prevalence of rickettsia exposure among dogs and cats in Bangkok and neighboring provinces. The samples from 367 dogs and 187 cats used in this study were leftover serum samples from routine laboratory testing stored at the Veterinary Teaching Hospital. In-house Enzyme-linked immunosorbent assay (ELISA) tests included IgG against the scrub typhus group (STG), typhus group (TG), and spotted fever group (SFG). The seroprevalence in pet dogs was 30.25% (111/367), including 21.53% for STG, 4.36% for TG, and 1.09% for SFG. Co-seroprevalence consisted of 2.72% for STG and TG, 0.27% for STG and SFG, and 0.27% for pangroup infection. The prevalence in cats was 62.56% (117/187), including 28.34% for STG, 4.28% for TG, and 6.42% for STG. Co-seroprevalence in cats consisted of STG and TG (4.28%), STG and SFG (5.35%), TG and SFG (3.21%), and three-group infection (10.69%). No significant difference in seroprevalence for the three serogroups was observed in any of the 64 districts sampled. The mean hematocrit level significantly decreased in seropositive dogs (P<0.05). Seropositive dogs and cats were detected in significantly greater numbers of anemia cases than nonanemia cases (P<0.05) (odds ratio: 7.93, 0.44, p = 0.00, p = 0.01). A significantly higher number of seropositive cats had decreased hemoglobin levels (P<0.05) (odds ratio: 3.63, p = 0.00). The seropositive samples significantly differed among older cats (P<0.05). These high exposures in pet dogs and cats could constitute important relationship dynamics between companion animals and rickettsial vectors. Significantly decreased hematocrit and hemoglobin levels indicated anemia in the exposed dogs and cats. The study findings will raise awareness of this neglected disease among pet owners and veterinary hospital personnel and aid in future public health preventative planning.

## Introduction

Rickettsiosis is a vector-borne zoonotic disease caused by arthropod-borne zoonotic intracellular bacteria. These bacteria reside in blood-sucking arthropods such as fleas, ticks, lice, and mites. Many rodents, dogs, cats, cattle, and wild animals are natural hosts for these blood-sucking arthropods [1]. The most common types of rickettsioses are caused by rickettsial organisms that fall within 3 serogroups: the scrub typhus group (STG), typhus group (TG) and spotted fever group (SFG) [2, 3]. Scrub typhus group organisms are from the genus *Orientia*, and both typhus and spotted fever group organisms are from the genus *Rickettsia*. Rickettsiosis is a prominent cause of sickness and mortality in Southeast Asia. Rickettsiosis is the fourth leading cause of fever in Southeast Asian returnees to countries on six continents, after malaria, dengue fever, and mononucleosis. After dengue fever, rickettsiosis is the second most often reported infection for nonmalarial fever in Southeast Asia [4–8].

*Rickettsia* spp. have been found in diverse animals in Thailand. For example, *Orientia tsutsugamushi* was found in rodents by PCR detection. *O*. *tsutsugamushi* DNA was also found in the mite genera *Blankaartia*, *Ascoschoengastia*, *Lorillatum*, and *Gahrliepia* [9, 10]. *Rickettsia felis* was found in cat fleas, *Ctenocephalides felis*, and *Ctenocephalides orientis* [11, 12]. In Thailand, *R*. *rickettsii*, *R*. *canada*, and *R*. *prowazekii* were found in dogs [13]. The seroreactivity of the spotted fever group was demonstrated using an enzyme-linked immunosorbent test in cats in northeastern Thailand [14]. Ticks, fleas and chiggers were collected from dogs and rats in southern Thailand, and DNA sequencing indicated that the species were *Rickettsia*. *asembonensis*, *Rickettsia* spp. *cf1*, *5*, and *O*. *tsutsugamushi* strain TA763 [15]. Molecular evidence confirmed *Rickettsia* spp. infection in rats caught from a Bangkok park. The confirmed species were *R*. *typhi* and *R*. *felis*, and this was the first report of *R*. *felis* DNA in rodents in Southeast Asia [16].

Rickettsiosis is classified as a neglected tropical disease (NTD), a group of infectious diseases that cause significant morbidity and mortality but receive little to no focus in public or veterinary health policies and support. However, NTDs continue to impact over a billion people living in underdeveloped or developing tropical countries, including those in Southeast Asia, Africa, and South America [17–19]. Therefore, diagnosis, treatment, and vaccine development are limited, and development of treatment is delayed. People in tropical countries are unaware of these diseases that harm their health. People are raising more pets as the social structure, family, and way of life have changed [20, 21]. Dogs and cats are now treated as family members rather than as “pets,” resulting in the “pet humanization” phenomenon. Awareness and comprehension of the importance of zoonoses are critical for society. The closer relationships between pets and owners have potentially increased the risk of disease transmission [22–24]. However, there are limited studies on the seroprevalence of rickettsiosis in pet animals in Thailand.

This study investigated the serological prevalence of rickettsiosis in pet dogs and cats in Bangkok and neighboring provinces. This research provides critical information for anyone at risk of these diseases, such as dog and cat owners, veterinary hospital staff, and relevant public health professionals.

## Materials and methods

### Ethics statement

The Animals for Scientific Purposes Act (A.D. 2015) of Thailand was followed, and authorization was acquired from the Laboratory Animal Committee of Kasetsart University’s Faculty of Veterinary Medicine (approval ID: ACKU63-VET-038). The author of this study was granted permission to use animals for scientific purposes (approval number U1-00325-2558).

### Sample collection

**Fig 1:**
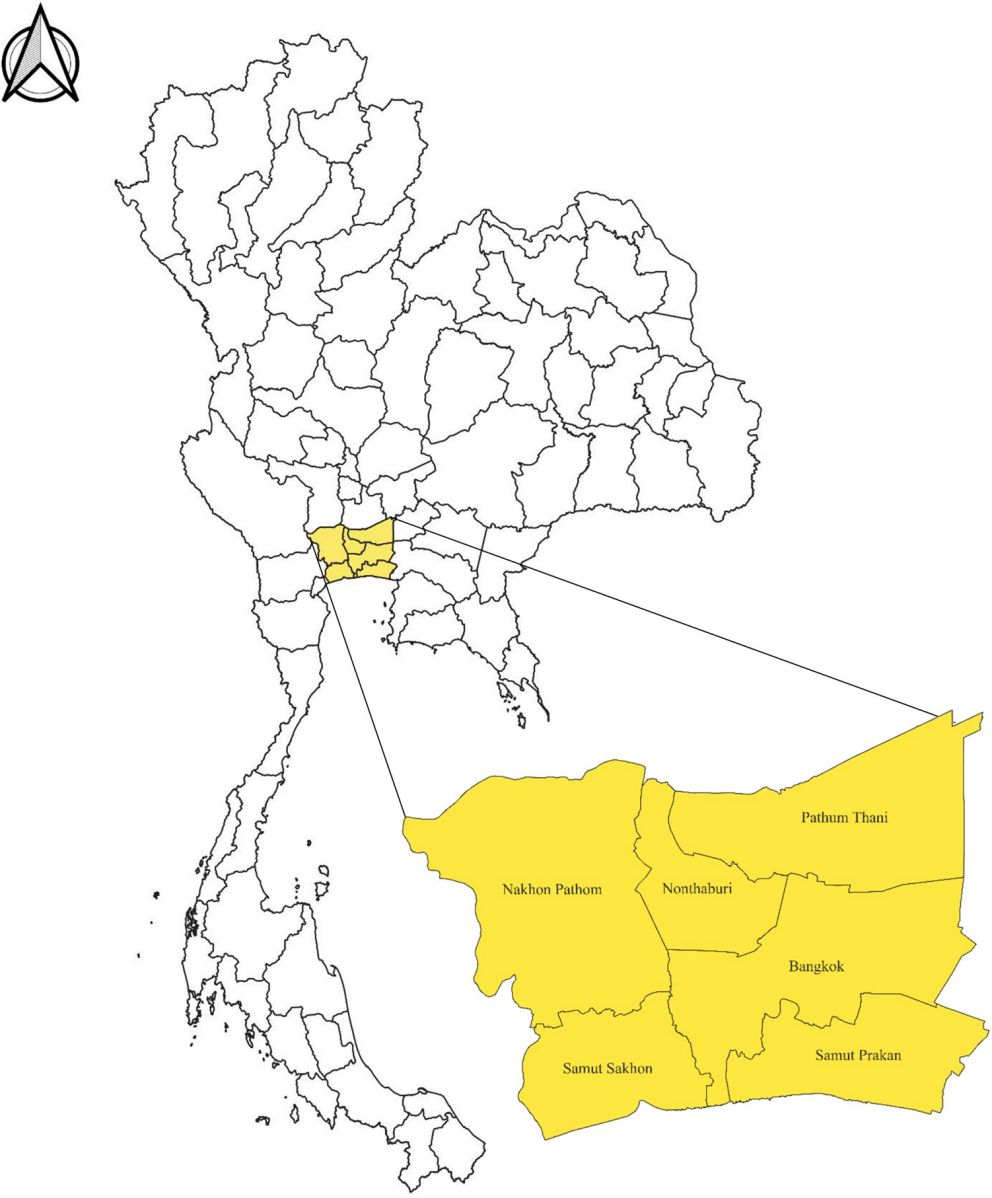
A map of the Bangkok metropolitan area where the study participants (cats and dogs) were from (the green color is the area of residence of the dogs and cats used in this study) [25].

All dog and cat whole blood and serum samples were randomly collected from leftover routine samples at the hematology laboratory, Kasetsart Veterinary Teaching Hospital, Faculty of Veterinary Medicine, Kasetsart University. The serum samples came from 367 dogs and 187 cats with nonspecific diseases. The serum samples were also selected at random. Only pet dogs and cats from Bangkok and adjacent provinces, including Nonthaburi, Samut Prakan, Pathum Thani, Samut Sakhon, and Nakhon Pathom, were collected for testing (Fig 1). Whole blood and serum were kept at -20°C until they were tested (Table 1).

**Table 1:** Summary of samples collected from pet dogs and cats by province.

Provinces	Pet dogs	Pet cats
Bangkok	217	106
Nonthaburi	66	37
Samut Prakan	28	13
Pathum Thani	51	27
Samut Sakhon	4	2
Nakhon Pathom	1	2
**Total**	**367**	**187**

### Enzyme-linked immunosorbent assay (ELISA)

The Mahidol-Oxford Tropical Medicine Research Unit (MORU) developed and produced the scrub typhus, typhus, and spotted fever antigens used in this investigation. The ELISA method used has been published previously [26–29].

The protocol, in brief, utilized 96-well plates coated with *O*. *tsutsugamushi* antigen (4 strains; *Karp*, *Kato*, *Gilliam*, and *TA716*), *R*. *typhi* antigen, and spotted fever group antigens (2 strains; *R*. *conorii* and *R*. *honei*). Horseradish peroxidase (HRP)-conjugated goat anti-dog/cat IgG (Sigma‒Aldrich, Germany) was used as the secondary antibody, and tetramethylbenzidine (TMB) (Thermo Scientific, USA) was used as the substrate. Plates were read at 450 nm (minus a reference value at 620 nm) (Bio-Rad iMark™ Microplate Absorbance Reader, USA). If a sample had an ELISA IgG nett OD greater than or equal to 0.5, it was considered positive for the presence of antibodies.

### Statistical analysis

We studied the association between seropositive samples of *Rickettsia* spp. in dogs and cats by using statistics to categorize the data based on factors that we believed would be linked to positive samples. To classify the data, we used information from the animal database of Kasetsart Veterinary Teaching Hospital. The data were divided into animal habitat, age, sex, breed, and hematology, which we used to segment the data for statistical analysis (S1 File). For the continuous variables (hematology: HCT, RBC, WBC, HGB, MCV, MCHC, MCH, PLT), we obtained descriptive statistics such as the means, medians, standard deviations (SDs), minimums, and maximums. We used the completed classification data for statistical analysis and tested the variables of interest as risk factors using the chi-square test based on the recorded data characteristics. R version 4.2.3 and NCSS 2023 version 23.0.1 were used to statistically analyze the general and hematological data and perform chi-square and odds ratio analyses. We conducted all statistical analyses with 95% confidence intervals, and we considered a p value < 0.05 significant.

## Results

The overall prevalence was 30.25% (111/367) in pet dogs. Of 367 pet dogs, 21.53% (79/367) were positive for the scrub typhus group. The typhus group seroprevalence was 4.36% (16/367), and the spotted fever group seroprevalence was 1.09% (4/367). Some dogs tested positive for more than one antigen. The codetection rate of scrub typhus and typhus was 2.72% (10/367). One dog was seropositive for both the scrub typhus and spotted fever groups (0.27%; 1/367). Multiple seropositivity was detected in a dog that tested positive for all three antigen groups, with 0.27% (1/367) (Table 2).

**Table 2:** Seroprevalence results of ELISA for dogs and cats.

Group of diseases	Seropositivity	
	Dogs	Cats
	(n = 367)	(n = 187)
Scrub typhus	79 (21.53%)	53 (28.34%)
Typhus	16 (4.36%)	8 (4.28%)
Spotted fever	4 (1.09%)	12 (6.42%)
Codetection of scrub typhus and typhus	10 (2.72%)	8 (4.28%)
Codetection of scrub typhus and spotted fever	1 (0.27%)	10 (5.35%)
Codetection of typhus and spotted fever	-	6 (3.21%)
Codetection of scrub typhus, typhus, and spotted fever	1 (0.27%)	20 (10.69%)
**Total**	**111 (30.25%)**	**117 (62.56%)**

In cats, the overall seroprevalence was 62.56% (117/187). A total of 28.34% (53/187) were positive for the scrub typhus group. The typhus group seroprevalence was 4.28% (8/187), and the spotted fever group seroprevalence was 6.42% (12/187). Additionally, some cats tested positive serologically for more than one antigen. Codetection of scrub typhus and typhus groups was 4.28% (8/187). The seroprevalence of the scrub typhus and spotted fever groups was 5.35% (10/187). The seroprevalence of the typhus and spotted fever groups was 3.21% (6/187). We also found that 10.69% of cats (20/187) tested positive serologically for all three groups of antigens (Table 2).

To investigate the correlation between urban and suburban areas, we divided all pet habitats into two categories: pets in Bangkok and pets in surrounding provinces. We performed a chi-square analysis to find the association between serological results and habitats. The results revealed no statistically significant difference in the prevalence of dogs and cats between Bangkok’s urban and suburban areas and its surrounding provinces (p value = 0.75 for the dog samples and p value = 0.84 for the cat samples) (Table 3).

**Table 3:** Statistical analysis of the number of dogs and cats by area using the chi-square test.

Species	Area	ELISA Results			χ^2^	p value
		Seropositive	Seronegative	Total		
**Dog**	Bangkok	67	150	217	0.10	0.75
	Non-Bangkok	44	106	150		
	Total	111	256	367		
**Cat**	Bangkok	67	39	106	0.04	0.84
	Non-Bangkok	50	31	81		
	Total	117	70	187		

The analysis used chi-squared independence tests to examine the relationship between each cat and dog age group. Three different age groups were categorized to find the association between age and serological outcome, including dogs and cats younger than one year, between 1 and 7 years, and older than seven years. The analysis results in the dog population showed no significant difference between the ages of dogs and serological results (p value = 0.80). In contrast to the results for dogs, significant differences were found in the cat population between the age groups (chi-square statistic of 19.73 and a p value = 0.00), indicating statistical significance at p < 0.05. The seropositive samples differed significantly among older cats (Table 4).

**Table 4:** Statistical analysis of the number of dogs and cats by age using the chi-square test.

Species	Area	ELISA Results			χ^2^	p value
		Seropositive	Seronegative	Total		
**Dog**	< 1 year	1	1	2	0.45	0.80
	1–7 years	16	35	51		
	>7 years	93	221	314		
	Total	110	257	367		
**Cat**	< 1 year	2	9	11	19.73	0.00*
	1–7 years	54	44	98		
	>7 years	61	17	78		
	Total	117	70	187		

* P < 0.05 using Pearson’s chi-square test.

The sex-based analysis was conducted using a chi-square test of independence to investigate the association between rickettsia exposure in males and females. Our findings indicated no significant difference between males and females regarding rickettsia exposure in dogs or cats, with p values = 0.80 and 0.81, respectively.

We collected data on the complete blood count (CBC), which measures the quantity and characteristics of three types of blood cells: red blood cells, white blood cells, and platelets. The data included values for hematocrit (HCT), red blood cell (RBC), white blood cell (WBC), hemoglobin (HGB), mean corpuscular volume (MCV), mean corpuscular hemoglobin concentration (MCHC), mean corpuscular hemoglobin (MCH), and platelet count (PLT) (Tables 5 and 6). These blood parameters were compared between seropositive and seronegative dogs and cats. The mean hematocrit (HCT) significantly decreased in seropositive dogs (P<0.05).

**Table 5:** Complete blood count (CBC) values of dog samples.

Parameters	Observed Range		Mean ± Standard Deviation (SD)		Normal Range [30]
	Seropositive	Seronegative	Seropositive	Seronegative	
HCT (%)	19.00–57.50	31.10–56.60	40.24 ± 8.04*	43.13 ± 4.77	35.00–57.00
RBC (10^6^/mcL)	2.36–9.33	3.30–9.81	6.41 ± 1.33	6.70 ± 1.12	4.95–7.87
WBC (10^3^/mcL)	1.39–38.26	2.20–39.26	12.60 ± 6.26	12.37 ± 5.71	5.00–14.10
HGB (g/dL)	6.00–21.30	7.00–19.80	14.31 ± 3.08	13.65 ± 2.38	11.90–18.90
MCV (fL)	38.77–80.51	38.45–80.51	63.26 ± 5.19	63.01 ± 5.49	66.00–77.00
MCHC (g/dL)	4.98–38.10	31.01–38.94	35.19 ± 3.21	35.45 ± 1.41	32.00–36.30
MCH (pg)	13.72–26.20	13.50–28.83	22.42 ± 2.01	22.47 ± 1.93	21.00–26.20
PLT (10^3^/mcL)	1.00–950.00	10.00–820.00	335.19 ± 176.13	350.23 ± 163.81	211.00–621.00

**Table 6:** Complete blood count (CBC) values of cat samples.

Parameters	Observed Range		Mean ± Standard Deviation (SD)		Normal Range [30]
	Seropositive	Seronegative	Seropositive	Seronegative	
HCT (%)	14.40–59.80	31.74–56.40	31.17 ± 7.77	31.74 ± 8.07	30.00–45.00
RBC (10^6^/mcL)	2.73–14.03	3.00–11.53	7.56 ± 2.13	7.59 ± 2.00	5.00–10.00
WBC (10^3^/mcL)	2.42–79.00	2.64–41.20	14.63 ± 11.19	13.24 ± 8.13	5.50–19.50
HGB (g/dL)	4.60–18.30	6.00–18.10	11.30 ± 2.97	12.16 ± 2.59	9.80–15.40
MCV (fL)	29.75–68.00	28.15–60.53	42.09 ± 6.35	42.77 ± 6.60	39.00–55.00
MCHC (g/dL)	29.85–39.94	29.20–35.69	36.10 ± 2.10	35.31 ± 2.04	30.00–36.00
MCH (pg)	10.86–25.08	10.28–22.98	15.20 ± 2.17	15.23 ± 1.94	13.00–17.00
PLT (10^3^/mcL)	44.00–614.00	98.0–700.00	273.99 ± 122.55	288.25 ± 117.05	300.00–800.00

We conducted a chi-squared independence test using all hematological data to investigate the association between complete blood count and exposure to *Rickettsia* spp. The analysis revealed a significant association between hematocrit and *Rickettsia* spp. exposure in dogs and cats (p < 0.05) (Table 7). The chi-square statistic of hemoglobin in cats was 14.01 with a p value of 0.00, which was significant at p < 0.05 (Table 8). Therefore, we found that the dog HCT, cat HCT, and cat HGB data showed significant associations between these blood parameters in dogs and cats and *Rickettsia* spp. exposure.

**Table 7:** Statistical analysis of ELISA results for dog samples by complete blood count (CBC) values using the chi-square test.

Parameters		ELISA Results			χ^2^	p value
		Seropositive	Seronegative	Total		
HCT	≥35%	87	230	317	30.31	0.00*
	<35%	24	8	32		
	Total	111	238	349		
RBC	≥4.95 (10^6^/mcL)	86	196	282	1.16	0.28
	<4.95 (10^6^/mcL)	25	42	67		
	Total Total	111	238	349		
WBC	5.00–14.10 (10^3^/mcL)	75	158	233	0.05	0.83
	>14.10 (10^3^/mcL)	36	80	116		
	Total	111	238	349		
HGB	≥11.90 (g/dL)	85	181	266	0.00	0.97
	<11.90 (g/dL)	26	56	82		
	Total	111	237	348		
MCV	Normal range	24	48	72	0.09	0.77
	Abnormal range	87	189	276		
	Total	111	237	348		
MCHC	Normal range	77	152	229	0.92	0.34
	Abnormal range	34	85	119		
	Total	111	237	348		
MCH	Normal range	93	201	294	0.03	0.87
	Abnormal range	18	37	55		
	Total	111	238	349		
PLT	≥211 (10^3^/mcL)	79	174	253	0.25	0.62
	<211 (10^3^/mcL)	32	62	94		
	Total	111	236	347		

* p < 0.05 using Pearson’s chi-square test.

**Table 8:** Statistical analysis of ELISA results for cat samples by complete blood count (CBC) values using the chi-square test.

Parameters		ELISA Results			χ^2^	p value
		Seropositive	Seronegative	Total		
HCT	≥30%	71	26	97	6.69	0.01*
	<30%	46	38	84		
	Total	117	64	181		
RBC	≥5.0 (10^6^/mcL)	87	49	136	0.11	0.74
	<5.0 (10^6^/mcL)	30	15	45		
	Total	117	64	181		
WBC	5.50–19.50 (10^3^/mcL)	71	49	120	0.04	0.85
	>19.50 (10^3^/mcL)	37	24	61		
	Total	108	73	181		
HGB	≥9.80 (g/dL)	58	50	108	14.01	0.00*
	<9.80 (g/dL)	59	14	73		
	Total	117	64	181		
MCV	Normal range	74	40	114	0.01	0.92
	Abnormal range	43	24	67		
	Total	117	64	181		
MCHC	Normal range	51	37	88	3.35	0.07
	Abnormal range	66	27	93		
	Total	117	64	181		
MCH	Normal range	92	50	142	0.01	0.94
	Abnormal range	25	14	39		
	Total	117	64	181		
PLT	≥300 (10^3^/mcL)	48	28	76	0.20	0.66
	<300 (10^3^/mcL)	69	35	104		
	Total	117	63	180		

* p < 0.05 using Pearson’s chi-square test.

Anemic dogs with HCT levels below 35% had a 7.93-fold greater chance of being seropositive than those with normal HCT levels (odds ratio: 7.93, p = 0.00). Anemic cats with HCT levels less than 30% had a 0.44-fold greater chance of being seropositive than those with normal HCT levels (odds ratio: 0.44, p = 0.01). For those cats with HGB levels below 9.8 g/dL, there was a 3.63-fold greater chance of being seropositive than those with normal HGB levels (odds ratio: 3.63, p = 0.00).

## Discussion

This study investigated the evidence of exposure of domesticated dogs and cats in the Bangkok Metropolitan Region (BMR) to *Rickettsia* spp. The findings indicated that all three disease groups (scrub typhus, typhus, and spotted fever) resulted in serological positivity using ELISA. This study is of great importance because it provides evidence of a high human exposure risk to *Rickettsia* spp. from pets. Moreover, this study revealed that many dogs and cats were exposed to *Rickettsia* spp., possibly due to their exposure to blood-sucking arthropods that may carry the bacteria. Previous reports have indicated detections of *Rickettsia* spp. in blood-sucking arthropods such as *Ctenocephalides felis felis*, especially in countries such as Thailand, the United States, and Bangladesh [31–33]. Additionally, there have been reports of cat fleas (*Ctenocephalides felis*) in northeastern Thailand that have tested positive for *R*. *asembonensis* [14], while ticks, fleas, and chiggers in southern Thailand have been found to harbor *O*. *tsutsugamushi* [16].

Vector-borne diseases (VBDs) were investigated in pet animals (dogs and cats) in Khukhot city, Pathum Thani Province, Thailand, and demonstrated a notable connection between age and the likelihood of infection among pets. The study specifically disclosed that adult cats are more vulnerable to VBDs than their younger counterparts [34]. In our research, older cats were also more at risk of exposure to *Rickettsia* spp. The higher prevalence among older cats might be due to their natural behavior of exploring the area surrounding their homes, as evidenced by a study conducted by the Central Tablelands Local Land Services (LLS) in New South Wales, Australia. The LLS used GPS to track the paths of domesticated cats and found that some cats roamed up to 3 kilometers away from their homes, including venturing into forests [35]. Keeping cats outdoors can expose them to various environmental factors, such as other animals, parasites, bacteria, viruses, and environmental hazards. For example, outdoor pets may come into contact with other animals, including strays or wild animals that could carry diseases such as rabies. Additionally, outdoor pets are at a higher risk of encountering parasites such as fleas, ticks, and mosquitoes, and outdoor cats are more likely to be infected with the parasites *Toxoplasma gondii* [36] and *Bartonella henselae* [37], which cause toxoplasmosis and cat scratch disease, respectively. Going outdoors is a significant risk factor for parasitic infections in domesticated cats, with outdoor cats being 2.77 times more likely to be infected with parasites than indoor cats [38]. It is important to note that keeping a cat outdoors can also pose a health risk to the owner [36, 37, 39–41].

One crucial factor to consider is the potential exposure of pet dogs and cats to *Rickettsia* spp., a disease that may be transmitted by rodents, particularly the oriental house rat (*Rattus tanezumi*), which has a widespread and adaptable habitat in all regions [42–44]. *Rattus rattus* is commonly found in public parks throughout Bangkok [16], in both urban and rural areas. Retrospective studies in Thailand have shown that rats, such as *Rattus tanezumi* in Nakhon Pathom Province, are immune to *O*. *tsutsugamushi*, *R*. *typhi*, and *R*. *honei* [45]. Furthermore, molecular evidence of *R*. *typhi* and *R*. *felis* has been found in small mammals captured from a park in Bangkok [16]. This information suggests that carrier animals and their vectors are ubiquitous in both urban and rural areas, and there is a possibility that these gnawing animals and their vectors may have close contact with pets, making this a matter of concern. Therefore, taking appropriate measures to protect pets from possible exposure to these vectors is important.

The statistical analysis of the hematologic data of pet dogs and cats found that *Rickettsia* spp. infections might lower the hematocrit (HCT) levels in dogs and cats, and hemoglobin (HGB) levels in seropositive cats were also significantly lower. *Rickettsia* infections involve the endothelial cells of the capillary wall, leading to damage and dysfunction of these cells [46, 47]. This damage results in anemia and lower hemoglobin levels in infected individuals. The mechanism by which *Rickettsia* infection causes anemia and lower hemoglobin levels is complex and not fully understood. However, these bacteria directly invade and damage the endothelial cells of the capillary wall, leading to the leakage of red blood cells and other blood components into the surrounding tissues. This leakage causes a decrease in the number of red blood cells and hemoglobin in the blood, resulting in anemia [47–49].

A study found numerous dogs and cats visiting veterinary hospitals testing positive for *Rickettsia* spp. These findings raise the question of whether veterinary staff are at risk of contracting the disease, especially given that previous reports have shown a significant prevalence among veterinary staff. A previous report from Ciudad Juarez, Chihuahua, Mexico, found a high prevalence of the disease among veterinary staff, with 21% of staff members at 63 veterinary clinics testing positive for *R*. *rickettsii*, and many staff reported a history of being bitten by the brown dog tick (*Rhipicephalus sanguineus*) [50]. The high prevalence of *Rickettsia* spp. in dogs and cats admitted to veterinary hospitals, combined with the risks to veterinary staff, underscores the importance of raising awareness and taking preventative measures to reduce the spread of the disease. Veterinary hospitals need to implement protocols to reduce the risks of infection, and staff must take precautions to protect themselves from exposure to *Rickettsia* spp.

The issue regarding cross-reaction is only relevant between Murine typhus and Spotted fever groups. It may be possible to discriminate further using the strength of the antibody response. It is difficult to discriminate between antibodies between the antigenic groups because of shared epitopes, and further work is required to characterize the immune response in animals and the levels of cross-reaction between the antigenic groups.

## Conclusion

This study provides serological evidence that pet dogs and cats in Bangkok and surrounding provinces are exposed to the following three diseases: scrub typhus, typhus, and spotted fever. These data imply that pet dogs and cats are frequently exposed to the disease and may constitute important reservoirs of rickettsiosis. Evaluating blood parameters, such as significantly decreased HCT and HGB levels, should be of concern in seropositive dogs and cats with various clinical signs. These blood parameters indicate anemia in exposed dogs and cats. However, these pets often show no specific signs of illness, and thus, it is difficult to monitor disease occurrence, if any. Hematological data derived from this study, such as hematocrit and hemoglobin data, were associated with infection. Therefore, the pathogenesis of rickettsiosis in dogs and cats should be further studied. This information may be helpful to veterinarians for preliminary screening of the causes of anemia in dogs and cats in tropical countries. Age-related exposure was also a major parameter. The study findings can provide basic information for those interested in using them as a guideline to monitor disease outbreaks in humans and pets. This study can also raise awareness of this neglected disease among pet owners and veterinary hospital personnel and aid in future public health preventative planning.

## Supporting information

S1 File Data on dogs and cats, including their age, sex, location, and all blood parameters. (XLSX)

## References

[1] Low VL, Tan TK, Khoo JJ, Lim FS, AbuBakar S. An overview of rickettsiae in Southeast Asia: Vector-animal-human interface. Acta Trop. 2020 Feb;202:105282. doi: 10.1016/j.actatropica.2019.105282 31778642

[2] Balcells ME, Rabagliati R, García P, Poggi H, Oddó D, Concha M, et al. Endemic scrub typhus-like illness, Chile. Emerg Infect Dis. 2011 Sep;17(9):1659–63. doi: 10.3201/eid1709.100960 21888791 PMC3322051

[3] Izzard L, Fuller A, Blacksell SD, Paris DH, Richards AL, Aukkanit N, et al. Isolation of a novel *Orientia* species (*O*. *chuto* sp. nov.) from a patient infected in Dubai. J Clin Microbiol. 2010 Dec;48(12):4404–9. doi: 10.1128/JCM.01526-10 20926708 PMC3008486

[4] Acestor N, Cooksey R, Newton PN, Ménard D, Guerin PJ, Nakagawa J, et al. Mapping the aetiology of non-malarial febrile illness in Southeast Asia through a systematic review—terra incognita impairing treatment policies. PLoS One. 2012;7(9):e44269. doi: 10.1371/journal.pone.0044269 22970193 PMC3435412

[5] Aung AK, Spelman DW, Murray RJ, Graves S. Rickettsial infections in Southeast Asia: implications for local populace and febrile returned travelers. Am J Trop Med Hyg. 2014 Sep;91(3):451–60. doi: 10.4269/ajtmh.14-0191 24957537 PMC4155544

[6] Freedman DO, Weld LH, Kozarsky PE, Fisk T, Robins R, von Sonnenburg F, et al. Cetron MS; GeoSentinel Surveillance Network. Spectrum of disease and relation to place of exposure among ill returned travelers. N Engl J Med. 2006 Jan 12;354(2):119–30. doi: 10.1056/NEJMoa051331 16407507

[7] Bottieau E, Clerinx J, Schrooten W, Van den Enden E, Wouters R, Van Esbroeck M, et al. Etiology and outcome of fever after a stay in the tropics. Arch Intern Med. 2006 Aug 14–28;166(15):1642–8. doi: 10.1001/archinte.166.15.1642 16908798

[8] Wilson ME, Weld LH, Boggild A, Keystone JS, Kain KC, von Sonnenburg F, et al. GeoSentinel Surveillance Network. Fever in returned travelers: results from the GeoSentinel Surveillance Network. Clin Infect Dis. 2007 Jun 15;44(12):1560–8. doi: 10.1086/518173 17516399

[9] Rodkvamtook W, Kuttasingkee N, Linsuwanon P, Sudsawat Y, Richards AL, Somsri M, et al. Scrub typhus outbreak in Chonburi Province, central Thailand, 2013. Emerg Infect Dis. 2018 Feb;24(2):361–365. doi: 10.3201/eid2402.171172 29350148 PMC5782907

[10] Takhampunya R, Korkusol A, Promsathaporn S, Tippayachai B, Leepitakrat S, Richards AL, et al. Heterogeneity of *Orientia tsutsugamushi* genotypes in field-collected trombiculid mites from wild-caught small mammals in Thailand. PLoS Negl Trop Dis. 2018 Jul 16;12(7):e0006632. doi: 10.1371/journal.pntd.0006632 30011267 PMC6062101

[11] Foongladda S, Inthawong D, Kositanont U, Gaywee J. Rickettsia, Ehrlichia, Anaplasma, and Bartonella in ticks and fleas from dogs and cats in Bangkok. Vector Borne Zoonotic Dis. 2011 Oct;11(10):1335–41. doi: 10.1089/vbz.2010.0174 21612535

[12] Phoosangwalthong P, Hii SF, Kamyingkird K, Kengradomkij C, Pinyopanuwat N, Chimnoi W, et al. Cats as potential mammalian reservoirs for *Rickettsia sp*. genotype RF2125 in Bangkok, Thailand. Vet Parasitol Reg Stud Reports. 2018 Aug;13:188–192. doi: 10.1016/j.vprsr.2018.07.001 31014872

[13] Suksawat J, Xuejie Y, Hancock SI, Hegarty BC, Nilkumhang P, Breitschwerdt EB. Serologic and molecular evidence of coinfection with multiple vector‐borne pathogens in dogs from Thailand. J Vet Intern Med. 2001 Sep-Oct;15(5):453–62. doi: 10.1892/0891-6640(2001)015&lt;0453:sameoc&gt;2.3.co;2 11596732

[14] Phomjareet S, Chaveerach P, Suksawat F, Jiang J, Richards AL. Spotted fever group Rickettsia infection of cats and cat fleas in Northeast Thailand. Vector Borne Zoonotic Dis. 2020 Aug;20(8):566–571. doi: 10.1089/vbz.2019.2564 32744925

[15] Sanprick A, Yooyen T, Rodkvamtook W. Survey of *Rickettsia* spp. and *Orientia tsutsugamushi* pathogens found in animal vectors (ticks, fleas, chiggers) in Bangkaew District, Phatthalung Province, Thailand. Korean J Parasitol. 2019 Apr;57(2):167–173. doi: 10.3347/kjp.2019.57.2.167 31104409 PMC6526216

[16] Rungrojn A, Chaisiri K, Paladsing Y, Morand S, Junjhon J, Blacksell SD, et al. Prevalence and molecular characterization of *Rickettsia* spp. from wild small mammals in public parks and urban areas of Bangkok metropolitan, Thailand. Trop Med Infect Dis. 2021 Nov 11;6(4):199. doi: 10.3390/tropicalmed6040199 34842856 PMC8628900

[17] Paris DH, Shelite TR, Day NP, Walker DH. Unresolved problems related to scrub typhus: a seriously neglected life-threatening disease. Am J Trop Med Hyg. 2013 Aug;89(2):301–7. doi: 10.4269/ajtmh.13-0064 23926142 PMC3741252

[18] Salje J, Weitzel T, Newton PN, Varghese GM, Day N. Rickettsial infections: A blind spot in our view of neglected tropical diseases. PLoS Negl Trop Dis. 2021 May 13;15(5):e0009353. doi: 10.1371/journal.pntd.0009353 33983936 PMC8118261

[19] Hotez PJ, Aksoy S, Brindley PJ, Kamhawi S. What constitutes a neglected tropical disease?. PLoS Negl Trop Dis. 2020 Jan 30;14(1):e0008001. doi: 10.1371/journal.pntd.0008001 31999732 PMC6991948

[20] Guo Z, Ren X, Zhao J, Jiao L, Xu Y. Can Pets Replace Children? The Interaction Effect of Pet Attachment and Subjective Socioeconomic Status on Fertility Intention. Int J Environ Res Public Health. 2021 Aug 15;18(16):8610. doi: 10.3390/ijerph18168610 34444359 PMC8394147

[21] Irvine L, Cilia L. More‐than‐human families: Pets, people, and practices in multispecies households. Sociology Compass. 2017;11(2):e12455. doi: 10.1111/soc4.12455

[22] Stull JW, Brophy J, Weese JS. Reducing the risk of pet-associated zoonotic infections. CMAJ. 2015 Jul 14;187(10):736–743. doi: 10.1503/cmaj.141020 25897046 PMC4500695

[23] Joosten P, Van Cleven A, Sarrazin S, Paepe D, De Sutter A, Dewulf J. Dogs and Their Owners Have Frequent and Intensive Contact. Int J Environ Res Public Health. 2020 Jun 16;17(12):4300. doi: 10.3390/ijerph17124300 32560155 PMC7345801

[24] Overgaauw PAM, Vinke CM, Hagen MAEV, Lipman LJA. A One Health Perspective on the Human-Companion Animal Relationship with Emphasis on Zoonotic Aspects. Int J Environ Res Public Health. 2020 May 27;17(11):3789. doi: 10.3390/ijerph17113789 32471058 PMC7312520

[25] Runfola D, Anderson A, Baier H, Crittenden M, Dowker E, Fuhrig S, et al. geoBoundaries: A global database of political administrative boundaries. PLoS One. 2020 Apr 24;15(4):e0231866. doi: 10.1371/journal.pone.0231866 ; PMCID: PMC7182183. 32330167 PMC7182183

[26] Elders PN, Dhawan S, Tanganuchitcharnchai A, Phommasone K, Chansamouth V, Day NP, et al. Diagnostic accuracy of an in-house Scrub Typhus enzyme linked immunoassay for the detection of IgM and IgG antibodies in Laos. PLoS Negl Trop Dis. 2020 Dec 7;14(12):e0008858. doi: 10.1371/journal.pntd.0008858 33284807 PMC7746293

[27] Phanichkrivalkosil M, Tanganuchitcharnchai A, Jintaworn S, Kantipong P, Laongnualpanich A, Chierakul W, et al. Determination of optimal diagnostic cut-offs for the Naval Medical Research Center scrub typhus IgM ELISA in Chiang Rai, Thailand. Am J Trop Med Hyg. 2019 May;100(5):1134–1140. doi: 10.4269/ajtmh.18-0675 30860022 PMC6493932

[28] Saraswati K, Phanichkrivalkosil M, Day NP, Blacksell SD. The validity of diagnostic cut-offs for commercial and in-house scrub typhus IgM and IgG ELISAs: A review of the evidence. PLoS Negl Trop Dis. 2019 Feb 4;13(2):e0007158. doi: 10.1371/journal.pntd.0007158 30716070 PMC6382213

[29] Suwanabun N, Chouriyagune C, Eamsila C, Watcharapichat P, Dasch GA, Howard RS, et al. Evaluation of an enzyme-linked immunosorbent assay in Thai scrub typhus patients. Am J Trop Med Hyg. 1997 Jan;56(1):38–43. doi: 10.4269/ajtmh.1997.56.38 9063359

[30] Fielder SE. MSD Veterinary Manual Rahway. New Jersey.USA Merck & Co., Inc. 2022 [updated Sep 2022]. Available from: https://www.msdvetmanual.com/special-subjects/reference-guides/hematology-reference-ranges#.

[31] Ahmed R, Paul SK, Hossain MA, Ahmed S, Mahmud MC, Nasreen SA, et al. Molecular detection of *Rickettsia felis* in humans, cats, and cat fleas in Bangladesh, 2013–2014. Vector Borne Zoonotic Dis. 2016 May;16(5):356–8. doi: 10.1089/vbz.2015.1886 26901499

[32] Assarasakorn S, Veir J, Hawley J, Brewer M, Morris A, Hill AE, et al. Prevalence of *Bartonella* species, hemoplasmas, and *Rickettsia felis* DNA in blood and fleas of cats in Bangkok, Thailand. Res Vet Sci. 2012 Dec;93(3):1213–6. doi: 10.1016/j.rvsc.2012.03.015 22521739

[33] Šlapeta Š, Šlapeta J. Molecular identity of cat fleas (*Ctenocephalides felis*) from cats in Georgia, USA carrying *Bartonella clarridgeiae*, *Bartonella henselae* and *Rickettsia* sp. RF2125. Vet Parasitol Reg Stud Reports. 2016 Jun;3–4:36–40. doi: 10.1016/j.vprsr.2016.06.005 31014497

[34] Luong NH, Kamyingkird K, Thammasonthijarern N, Phasuk J, Nimsuphan B, Pattanatanang K, et al. Companion Vector-Borne Pathogens and Associated Risk Factors in Apparently Healthy Pet Animals (Dogs and Cats) in Khukhot City Municipality, Pathum Thani Province, Thailand. Pathogens. 2023 Mar 1;12(3):391. doi: 10.3390/pathogens12030391 36986313 PMC10058879

[35] Pearce M. Cat tracking program makes owners re-think pets’ behaviour and how they manage their moggies Australia. ABC Central West. 2016 [updated 21 May 2016; cited 2023:5]. Available from: https://www.abc.net.au/news/2016-05-20/cat-tracking-program-makes-owners-re-think-pet-behaviour/7431248?utm_campaign=abc_news_web&utm_content=link&utm_medium=content_shared&utm_source=abc_news_web.

[36] Hill D, Dubey JP. *Toxoplasma gondii*: transmission, diagnosis and prevention. Clin Microbiol Infect. 2002 Oct;8(10):634–40. doi: 10.1046/j.1469-0691.2002.00485.x 12390281

[37] Chomel BB, Boulouis HJ, Maruyama S, Breitschwerdt EB. *Bartonella* spp. in pets and effect on human health. Emerg Infect Dis. 2006 Mar;12(3):389–94. doi: 10.3201/eid1203.050931 16704774 PMC3291446

[38] Chalkowski K, Wilson AE, Lepczyk CA, Zohdy S. Who let the cats out? A global meta-analysis on risk of parasitic infection in indoor versus outdoor domestic cats (*Felis catus*). Biol Lett. 2019 Apr 26;15(4):20180840. doi: 10.1098/rsbl.2018.0840 30991913 PMC6501354

[39] Fisher M. Toxocara cati: an underestimated zoonotic agent. Trends Parasitol. 2003 Apr;19(4):167–70. doi: 10.1016/s1471-4922(03)00027-8 12689646

[40] Lepczyk CA, Lohr CA, Duffy DC. A review of cat behavior in relation to disease risk and management options. Appl Anim Behav Sci. 2015;173:29–39. doi: 10.1016/j.applanim.2015.07.002

[41] Loyd KA, Hernandez SM, Abernathy KJ, Shock BC, Marshall GJ. Risk behaviours exhibited by free-roaming cats in a suburban US town. Vet Rec. 2013 Sep 28;173(12):295. doi: 10.1136/vr.101222 23913174

[42] Blasdell K, Bordes F, Chaisiri K, Chaval Y, Claude J, Cosson J-F, et al. Progress on research on rodents and rodent-borne zoonoses in South-east Asia. Wildl. Res. 2015;42(2):98–107.

[43] Kosoy M, Khlyap L, Cosson JF, Morand S. Aboriginal and invasive rats of genus Rattus as hosts of infectious agents. Vector Borne Zoonotic Dis. 2015 Jan;15(1):3–12. doi: 10.1089/vbz.2014.1629 25629775

[44] Pages M, Bazin E, Galan M, Chaval Y, Claude J, Herbreteau V, et al. Cytonuclear discordance among Southeast Asian black rats (*Rattus rattus* complex). Mol Ecol. 2013 Feb;22(4):1019–34. doi: 10.1111/mec.12149 23278980

[45] Prompiram P, Poltep K, Pamonsupornvichit S, Wongwadhunyoo W, Chamsai T, Rodkvamtook W. Rickettsiae exposure related to habitats of the oriental house rat (*Rattus tanezumi*, Temminck, 1844) in Salaya suburb, Thailand. Int J Parasitol Parasites Wildl. 2020 Aug 1;13:22–26. doi: 10.1016/j.ijppaw.2020.07.015 32793413 PMC7415620

[46] Valbuena G, Walker DH. Infection of the endothelium by members of the order Rickettsiales. Thromb Haemost. 2009 Dec;102(6):1071–9. doi: 10.1160/TH09-03-0186 19967137 PMC2913309

[47] Walker DH, Ismail N. Emerging and re-emerging rickettsioses: endothelial cell infection and early disease events. Nat Rev Microbiol. 2008 May;6(5):375–86. doi: 10.1038/nrmicro1866 18414502

[48] Gottlieb M, Long B, Koyfman A. The evaluation and management of Rocky Mountain spotted fever in the emergency department: a review of the literature. J Emerg Med. 2018 Jul;55(1):42–50. doi: 10.1016/j.jemermed.2018.02.043 29685474

[49] Tsioutis C, Zafeiri M, Avramopoulos A, Prousali E, Miligkos M, Karageorgos SA. Clinical and laboratory characteristics, epidemiology, and outcomes of murine typhus: a systematic review. Acta Trop. 2017;166:16–24. doi: 10.1016/j.actatropica.2016.10.018 27983969

[50] Escarcega-Avila ALM, Jiménez-Vega F, Quezada-Casasola A, Mora-Covarrubias A. Serologic evidence of rickettsial diseases associated with tick bites in workers of urban veterinary clinics. J Vector Borne Dis. 2020 Jan-Mar;57(1):40–46. doi: 10.4103/0972-9062.308799 33818454

